# Research Trends and Geographical Contribution in the Field of Perinatal Mental Health: A Bibliometric Analysis from 1900 to 2020

**DOI:** 10.1089/whr.2021.0144

**Published:** 2022-07-25

**Authors:** Usman Ali, Ahmed Waqas, Muhammad Ayub

**Affiliations:** ^1^ Academic Department of Psychiatry and Behavioral Sciences, King Edward Medical University/Mayo Hospital, Lahore, Pakistan.; ^2^ Institute of Population Health, University of Liverpool, United Kingdom.; ^3^ Department of Psychiatry, University College London, London, England.

**Keywords:** perinatal mental health, bibliometrics, postnatal

## Abstract

**Objective:**

The object of this research is to identify growth and geographical distribution of research in the field of perinatal mental health and key research areas.

**Materials and Methods:**

We performed a bibliometric analysis on research documents on perinatal mental health from 1900 to 2020. Web of Science was used to download bibliometric data. Search strategy used generic index terms with specific free text searches using “AND” as Boolean function. For example, psycho AND perinatal. Microsoft Excel was used to identify research growth, geographical and research institutes contribution to research in the field. Citation analysis was done using VOSviewer.

**Results:**

A total of 11,081 articles were extracted. The most cited research was by Cox et al., which introduced Edinburgh Postnatal Depression Scale. There was exponential increase in the research documents from 1990 to 2020. However, most of the research was contributed by the global north. Among emerging countries, India, South Africa, and Brazil did not contribute in the perinatal research. Postnatal depression and its risk factors were most studied themes. Paternal perinatal mental health and impact on mental health of children were understudied themes.

**Conclusion:**

Perinatal mental health research outpaced growth in biomedical research in the past 30 years. The work of leading researcher in the field was initially inspired by his experience in Uganda encountering postnatal depression. However, even after half a century, there is still less contribution from developing countries. This analysis highlights research and possibly access to services inequities in developing countries.

## Background

Bibliometric analysis is a type of scientometric analysis that aims at knowledge mapping by using bibliographic data in a certain field. The bibliometric analysis helps identify the research growth, trends, and productivity over a period in a field of interest.^1–4^ Through bibliometric analysis, we can identify influential articles, authors, journals, and countries in the field of interest. It also provides insight to the scientific community on most researched themes and thus helps identify knowledge gaps.^1–4^The information obtained from bibliometric analysis thus guides the researchers and research institutes to fill in those gaps by further research.

The bibliometric analysis also informs the scientific journals, research institutes, and universities of their scientific productivity through indicators such as documents contributed and citations received by their work in the field over a period.^3,4^ With knowledge mapping, we can highlight geographical disparities in research, the collaborations between research groups and countries. By informing the scientific fraternity and knowledge users such as donors, policymakers, and governments can help promote the knowledge creation and use.

Pregnancy has psychological and mental health implications for both mother and her family, including both father and children, and affects the family bonding. The research evidence is quite robust to show that mothers are at an increased risk of common mental illnesses. This includes depression, psychosis, and anxiety disorders. The incidence of postnatal depression is up to 12% and prevalence of 17%. The prevalence of antenatal anxiety is 15%–20%, whereas postnatal anxiety is 10%.^5^ There is also an increased relapse rate in women with pre-existing mental illness. Howard and Khalifeh state that 1 or 2 out of 1000 women may require inpatient psychiatric care, which shows that many women may suffer compromised quality of life after giving birth.^5,6^

Fathers may also suffer from mental illness such as depressive disorder, although there is less research in this area.^7^ Research shows poor maternal and paternal mental health in the perinatal period predisposes children to behavioral problems, anxiety, and depression.^5,8,9^ It is posited that 75% of perinatal mental illness economic burden is contributed by long-term child morbidity.^5^ Many researchers have examined risk factors for poor perinatal mental health. Lower socioeconomic status, lack of social support, lack of empowerment, perceived stress for the desired gender of child, and ethnicity are some of the identified risk factors for perinatal depression, anxiety, and sleep disturbances.^5,10,11^ Researchers have also highlighted that health disparities exist in perinatal mental health.^5,10,11^

Despite the importance of perinatal mental health, the research base seems to have particularly focused on postnatal depression. Furthermore, the large volume of research in scientific fields is contributed by the global north. There is substantial evidence that the perinatal mental health problems may differ in disease burden, risk factors, and characteristics among low- and middle-income countries (LMICs) as compared with high-income countries (HICs).^5,12,13^

Hence, it is important to undertake this bibliometric analysis to identify geographical variation in terms of research productivity, highlight areas of extensive research, research gaps in extensively researched topics, and under-researched areas. The article aims to provide an insight in perinatal mental health research through bibliometric analysis.

## Materials and Methods

### Data collection

Bibliographic data of articles related to perinatal mental health was collected from Web of Science (WoS) until December 2020. Key search words used were depress* OR anxiety OR anxious OR trauma* OR psycho* OR mental OR psycholo* OR psychiatry* AND perinatal OR pregnan* OR prenatal OR postnatal OR prepartum OR postpartum OR antepartum OR peripartum. Bibliometric data were downloaded in batches of 500 that is, the maximum that WoS allows. Seventeen data files were downloaded using this strategy.

### Data validation

To check the validly of our data representing our field of interest we used two strategies. In the first method, top 144 articles, which were cited >200 times, were manually searched on WoS and their abstracts were reviewed. In the second method, abstracts of top five cited articles in bibliographic data from each 5-year slice between 1980 and 2020 were reviewed. Both the methods confirmed that our bibliographic data consisted of articles on perinatal mental illnesses and health as we defined in our study objectives.

### Data analysis

For analyzing data, VOSviewer was used. VOSviewer is an open access Java-based software tool for visualizing networks in bibliographic data.^14^ Data pertaining to the top organizations, authors, countries, and journals were imported from VOSviewer to Microsoft Excel where frequency and distributions were computed. Year-wise publication trend was analyzed and graphs were generated. For generating an interactive map based on documents and citations of each country, StatPlanet was used. StatPlanet is an open access tool for generating maps and graphs (https://www.statsilk.com/software/statplanet).

For network visualization, we have used VOSviewer. Citation analysis was done with “country” set as unit of analysis. A cutoff at least five documents and five citations were chosen to run citation analysis.

To visualize clusters of thematic areas of perinatal mental health research we have included top 100 key words (all) in network visualization using VOSviewer and later reduced them to top 50 and 25 words. Since, the theoretical clusters remained the same when reducing the number of key words to 25 we chose top 100 key words to gain in-depth knowledge about dimensions of each cluster. Approval of ethical review was waived off by the institutional review board of King Edward Medical University, Lahore.

## Results

**FIG. 1.**
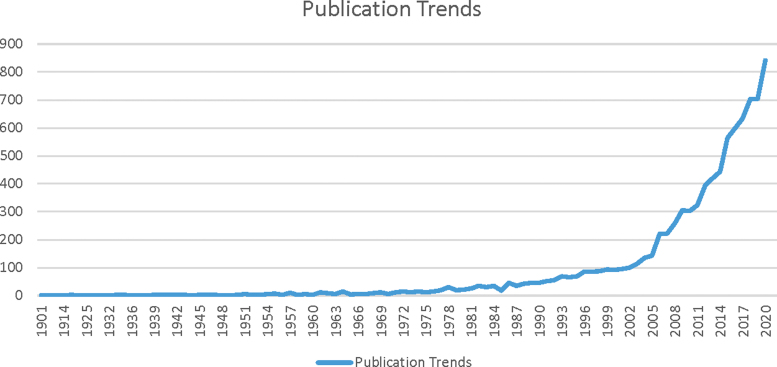
Publication trends in field of perinatal mental health from 1900 to 2020.

11,081 articles were extracted from WoS database. These articles were published from year 1900 to 2020 by 134 countries. This set of articles carried 291,244 citations (mean 26.2). Trend of publications is shown in Figure 1.

**FIG. 2.**
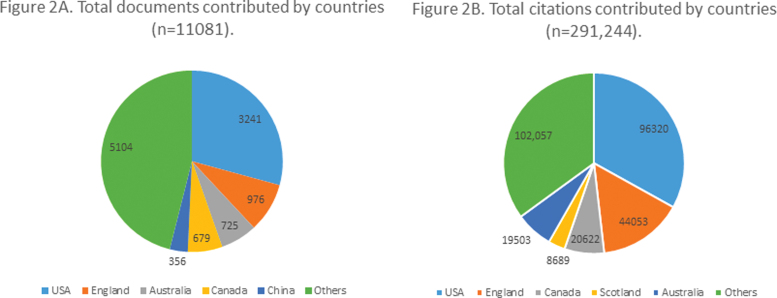
Countries with highest research productive (**A** shows countries contributing most documents; **B** shows countries whose documents received most citations).

Of countries contributing, 81 (60.4%) countries had at least five documents and five citations (total). They contributed 10,987 documents (99.15%), with 290,247 (99.6%) total citations. Top five countries with the greatest number of documents and citations for these documents contributed in field of common mental illnesses during perinatal period are listed in Figure 2.

United States, England, Canada, Australia, and China were top five countries contributing to literature on perinatal mental health. However, in list of top five countries with most citations, China was replaced by Scotland, which contributed to 72 research items and 8689 citations. Whereas China had 356 documents and only 4585 citations. China ranked at 11th place in top cited countries and was the top country outside United States and Europe in terms of research productivity in the world. The United States alone contributed to 32.25% of citations, 29.4% of documents, and 20.6% of total-link strengths.

**FIG. 3.**
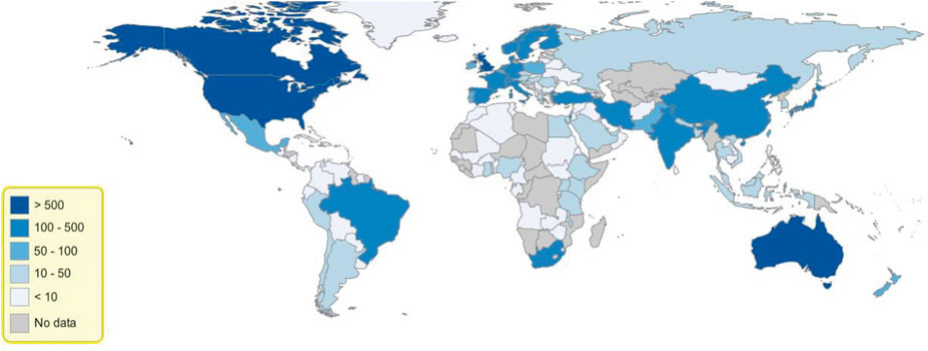
World map based on documents contributed by each country.

We also produced a world map where countries were represented in shades of blue, the intensity of which pertains to the number of documents contributed by the country (Fig. 3). The world map generally shows that countries from Eastern Europe, Central Asia, Middle East, Africa, and South America (with exception of Brazil and South Africa) have contributed less in the field of perinatal mental health research. World map based on citations received by countries showed similar trend and hence not included.

**FIG. 4.**
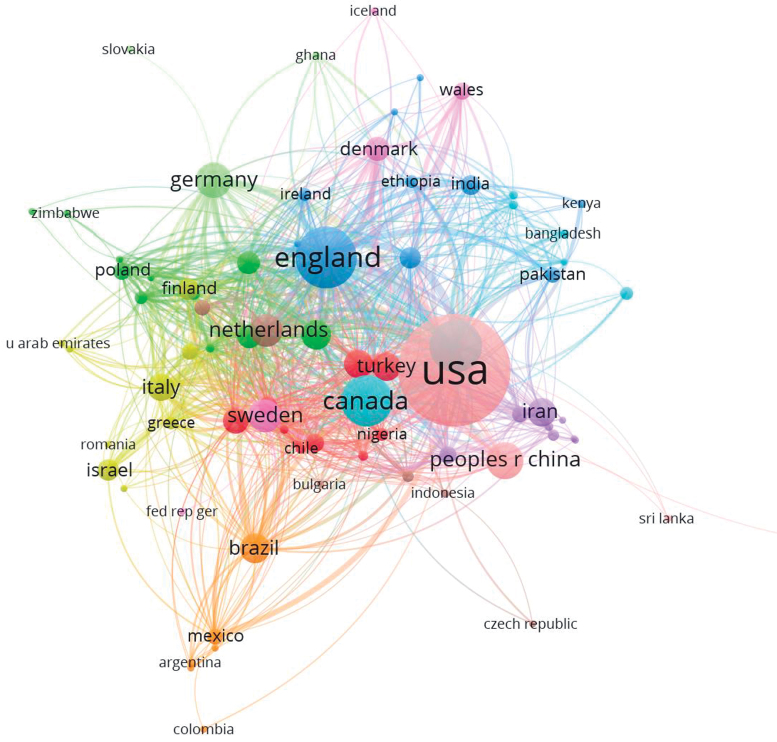
Citation analysis based on countries.

To explore collaboration among most cited countries, citation analysis was done as shown in Figure 4. In the citation analysis, the size of the country circle shows the research productivity and the lines joining countries show collaboration. Examining the citation analysis show that countries from Europe and the United States not only have high research productivity but also collaborate with one another. However, countries from Africa such has Ethiopia, Zimbabwe, and Slovakia have less research productivity and collaboration in research in perinatal mental health. The citation analysis shows that the involvement of countries from global south is lacking.

We have also conducted co-occurrence analysis of all key words from each document. The VOSviewer has identified 15,409 key words. One hundred one key words were used at least 115 times or more. List of top 100 most cited words are given as Supplementary Appendix SA1.

These key words can be arranged in four clusters.

1. The largest cluster here represented in red pertained to diagnostic labels such as depression, stress, mood disorders, and anxiety disorders.
2. The second cluster represented in green focused on psychometrics terms such as inventory, scale, validation, reliability, validity, and screening.
3. The third and fourth cluster represented in blue and purple are related to social aspects of research such as attitudes, experiences, support, care, and barriers. In overlay visualization, key words from this cluster appear in yellow, which means they are the words from recent publications.
4. Whereas the fourth cluster, represented in yellow is related to risk factors and impact of common mental illnesses occurring during perinatal period.

Each of the top 100 documents had >248 citations. Table 1 shows the top cited documents, their full title, journal of publication, and citations received.

**Table 1.** Articles with Most Citations in Research of Common Mental Illnesses in Perinatal Period

Sr. no.	Title	Journal (year)	No. of citations
1	Detection of postnatal depression. Development of the 10-item Edinburgh Postnatal Depression Scale^15^	*British Journal of Psychiatry* (1987)	6227
2	Rates and risk of postpartum depression—A meta-analysis^16^	*International Review of Psychiatry* (1996)	1957
3	Antenatal risk factors for postpartum depression: A synthesis of recent literature^17^	*General Hospital Psychiatry* (2004)	1001
4	Prenatal exposure to maternal depression, neonatal methylation of human glucocorticoid receptor gene (NR3C1), and infant cortisol stress responses^18^	*Epigenetics* (2008)	852
5	Cohort study of depressed mood during pregnancy and after childbirth^19^	*British Medical Journal* (2001)	811
6	The impact of postnatal depression and associated adversity on early mother–infant interactions and later infant outcome^20^	*Child Development* (1996)	789
7	The course of anxiety and depression through pregnancy and the postpartum in a community sample^21^	*Journal of Affective Disorders* (2004)	687
8	Effects of perinatal mental disorders on the fetus and child^12^	*The Lancet* (2014)	656
9	Low maternal free thyroxine concentrations during early pregnancy are associated with impaired psychomotor development in infancy^22^	*Clinical Endocrinology* (1999)	625
10	The impact of postnatal depression on infant development^23^	*Child Psychology & Psychiatry & Allied Disciplines* (1992)	625

Of 29,766 authors, 877 (2.95%) had five or more documents and 15,673 (52.6%) authors had 10 or more citations. Table 2 shows top 10 ranked authors for documents contributed and citations. Authors unique to either list are highlighted in gray.

**Table 2.** Authors Contributing to Most Documents and Citations in Field of Perinatal Mental Health

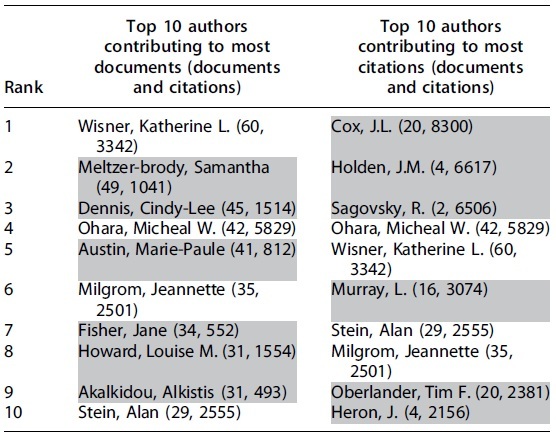

Of 6498 organizations, only 406 (6.24%) had >10 citations. Top ranking organization based on documents and citations is listed in Table 3. Organizations that could not make in both the lists are highlighted in gray.

**Table 3.** Organization Contributing to Most Documents and Citations

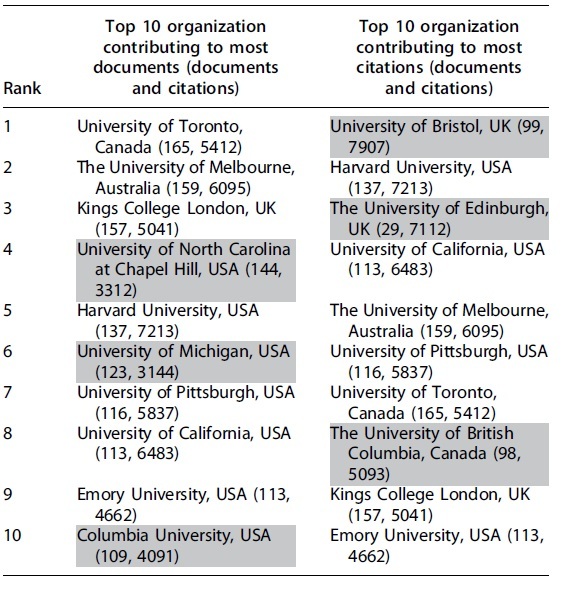

Of 1772 sources, 943 had 10 or more citations, 372 had more than five documents published in them. Journals unique to either list are highlighted in gray (Table 4).

**Table 4.** Top Ten Journals Contributing to Most Documents in Field of Perinatal Mental Health

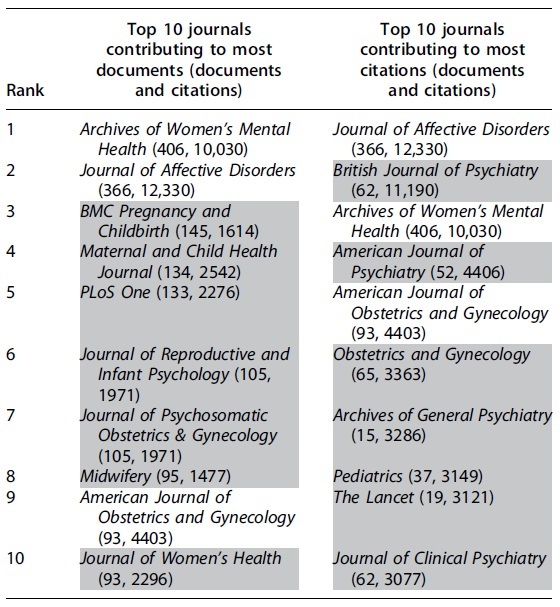

## Discussion

Our research shows that there has been a surge in the field of perinatal mental health research since the start of twenty-first century. The number of publications per year remained <50 till 1990, and by 2001 the number increased twofold. Compared with 2000 the number of publications per year was 3.2 times in 2010. The growth continued in the next decade (2010–2020) and compared with 2010 it increased by 2.7-fold in 2019. In a research article identifying trends of biomedical research across the globe, it was found that the increase in number of publications in biomedical sciences remained at a constant rate in 5-year cohorts from 1993 to 2012.^20^

The volume of research in 2012–2018 was roughly double of the research volume in 1993–1997 whereas, in perinatal mental health research we found 5.6 times increase during this time period, predominantly in the twenty-first century. This trend of rise in research on perinatal mental health goes along with an increase in research conducted over this time period in field of mental health.^21,22^ Larivière and Grant argued that the potential driver for increase in research in the field of mental health is because of increase in funding opportunities available for mental health research.^21^

The impetus for increase in perinatal mental health research was the study by Cox et al., which validated the Edinburgh Postnatal Depression Scale. The study was published in 1987 and till date is the most cited research article in the field of perinatal mental health.^24^ This research has provided researchers and clinicians a psychometric tool to identify and gauge postnatal depression and as a result there was a gradual increase in publications on perinatal mental health due to increased objective method for screening of this separate diagnostic entity and hence quantifying the burden of disease.

Among countries mostly contributing in perinatal mental health research in terms of documents contributed, United States lies at the top. Xu et al. have cited that United States is the leading contributor in biomedical research followed by China, United Kingdom, Japan, and Germany.^20^ There is also an increased trend of research in mental health in twenty-first century in BRICS countries that is, Brazil, Russia, India, China, and South Africa. China ranked fifth in perinatal mental health research. In our analysis, Brazil occupied 10th position, in terms of research productivity based on publications contributed. In terms of biomedical research, Brazil is ranked on 14th position.^20^

However, Russia, India, and South Africa did not have substantial contributions in perinatal mental health research. India, which represents a diverse population of South Asia, has been ranked as ninth top contributor in biomedical research from 1993 to 2012, but in the field of perinatal mental health research India occupied 24th rank.^23^ This may be a reflection of sociocultural factors where women's mental health receives less attention in Asian countries who otherwise contribute more to biomedical research. It is significant in this context that India did have increased number of publications in general mental health.^20^ The bibliometric analysis done on mental health research by Lariviere et al. reports a smaller number of publications contributed by Japan, France, Spain, South Korea, and Turkey on mental health as compared with research publications in other fields of biomedical sciences.

In our findings, Japan, France, Spain, and South Korea did not perform well on research contribution to perinatal mental health. However, Turkey did retain its rank at least when compared with biomedical research and perinatal mental health research. Australia, in contrast, lies at 12th rank in biomedical research but ranks 5th in perinatal mental health research.^20^ This is because the Australian government is cognizant of perinatal mental health needs and devised National Perinatal Mental Health Action Plan in 2008 for routine screening of perinatal mental health.^25^

As a result the percentage of women who were unscreened for mental health during pregnancy dropped from 40.6% to 1.7% from 2000 to 2017.^26^ Australia also has the largest women's health cohort study going on, research updates of which are frequently published and cited.^27,28^ In general, countries with more number of publications spend more on service delivery and community-based interventions for perinatal mental health, connecting research and translation of evidence into practice.^5,8^ Dr. Cox's initial work in Uganda inspired studies on maternal mental health, but 50 years later disparities between HICs and LMICs remain as stark as it was at that time.^29^

We have used an index of publications per million populations for top five countries contributing to most documents. The index is calculated by dividing number of publications by population in millions (in 2019) of countries. The index shows that Australia has 28.58 publications per million population, which is highest among top five countries. This is followed by Canada (18), United Kingdom (14.64), United States (9.87), and China (0.254). The top three documents producing institutes are located in Canada, Australia, and United Kingdom followed by U.S. universities in next seven ranks.

The top 10 institutes with most citations are, however, different. University of Bristol has fewer documents, but it is the most cited University across globe. University of Edinburgh made to the third rank because of influential article by Cox et al.^24^ Other than this article it has only 28 research articles to its credit. The research yield from Africa, Eastern Europe, Central Asia, Far East Asia, and Latin America with exception of Brazil was small. There are studies conducted on immigrants from these countries.^12,13^ However, this may not be representative population of the country of origin.

We have also searched for top five cited articles in 5 years cohort from 2001 to 2020. The time period was chosen as it represented bloom in publications; furthermore, it was broken in 5 years cohort to get granular data and identify recent trends. Of 20 articles identified only 1 studied depression in fathers, 5 studied effects on children of which 2 were systematic reviews. There was scarcity of research on paternal mental health and neurobiological aspects of perinatal mental illnesses. Network visualization using VOSviewer reveals that depression during or after pregnancy and associated risk factors were main research areas, whereas other perinatal mental health disorders were masked by research on the former. There is also lack of research on pharmacological treatment option specifically for perinatal mental illnesses,^7^ probably because of concerns about safety of medication during this period. Recently few randomized control trials are published, which explored role of probiotics in treatment of maternal anxiety and depression.^30–32^

Looking at the journals with most documents and citations the trend is interesting. As anticipated, journal on women health have most documents contributed on maternal mental health. Eight in top 10 journals with most documents belong to this category. However, only two of these journals rank in top 10 cited journals in mainstream.

## Conclusion

The bibliometric analysis on perinatal mental health informs us that the research and services disparity exist even 50 years after the initial work in the field. The general practitioners realized the importance of perinatal mental health because of their involvement with patients, but the academic psychiatrists' role was crucial in expanding the knowledge base over the years. This has translated in an increase in resource allocation by political governments and development of national plans for improved mental health services in HICs. LMICs continue to lag behind in research and service provision and future efforts may need to focus on addressing this imbalance through increased collaboration between HIC and LMIC. These collaborations can fill the gaps in funding and expertise.

## Supplementary Material

Supplemental data

## Supplementary Material

Supplementary Appendix SA1

## Abbreviations Used

BRICS: Brazil, Russia, India, China, and South Africa
HICs: high-income countries
LMICs: low- and middle-income countries
WoS: web of science
